# Repression of Dicer is associated with invasive phenotype and chemoresistance in ovarian cancer

**DOI:** 10.3892/ol.2013.1158

**Published:** 2013-01-28

**Authors:** YAN KUANG, JING CAI, DONGLIN LI, QIN HAN, JIN CAO, ZEHUA WANG

**Affiliations:** 1 Department of Obstetrics and Gynecology, Union Hospital, Tongji Medical College, Huazhong University of Science and Technology, Wuhan 430022;; 2 Department of Obstetrics and Gynecology, First Affiliated Hospital, GuangXi Medical University, Nanning 530021, P.R. China

**Keywords:** Dicer, ovarian cancer, cell proliferation, migration, cisplatin resistance, EZH2

## Abstract

Dicer is a key enzyme that processes microRNA (miRNA) precursors into their mature form, enabling them to regulate gene expression. However, the effects of Dicer on the biological behavior of cancer cells remain largely unclear. In this study, it was demonstrated that Dicer down-regulation promoted cell proliferation, migration and cell cycle progression in A2780 and SKOV3 ovarian cancer cells. Furthermore, Dicer expression was significantly decreased in cisplatin-resistant A2780 cells (A2780/DDP) compared with parental A2780 cells. Knockdown of Dicer by RNA interference decreased the sensitivity of A2780 cells to cisplatin. Moreover, EZH2 depletion by short hairpin RNA (shRNA) increased the expression of Dicer *in vitro*. Our data suggest that Dicer is involved in numerous biological/pathological processes, including drug resistance in ovarian cancer, and that its expression may be regulated by EZH2.

## Introduction

MicroRNAs (miRNAs) are small, endogenous non-coding RNA molecules (∼22 bases) that are capable of modulating the post-transcriptional regulation of numerous cellular genes (1). Regulated miRNA expression has been demonstrated in a variety of human cancer types, including chronic lymphocytic leukemia (2), lung cancer (3), colorectal neoplasia (4), and pancreatic endocrine and acinar tumors (5). Therefore, understanding the mechanisms that control miRNA expression in cancer and the functional consequences of this may provide novel insights into improvements in the classification, prognosis prediction and treatment of cancer.

Dicer is an essential member of the RNase III family, which controls maturation of miRNAs in the cytoplasm from miRNA precursors (pre-miRNAs) (6). As an upstream modulator of miRNAs, Dicer downregulation may promote cellular transformation and tumorigenesis via a global decrease in miRNA expression (7). It has been demonstrated that dysregulation of miRNAs is involved in the pathogenesis, tumor phenotype and chemosensitivity of ovarian cancer (8). Moreover, downregulation of Dicer is detectable in 60% of patients with invasive epithelial ovarian cancer and is associated with poor clinical outcomes (9). However, the role of Dicer in the biological behavior and chemosensitivity of ovarian cancer remains largely unclear.

Enhancer of zeste homolog 2 (EZH2), known as the catalytic submit of polycomb repressive complex 2 (PRC2), is a highly conserved histone methyltransferase that targets histone 3 lysine 27. EZH2 is recognized as a key epigenetic regulator associated with transcriptional repression and gene silencing (10). Taniguchi *et al* demonstrated that epigenetic silencing of Kruppel-like factor 2, which generally acts as a tumor suppressor in cancer through direct transcriptional repression, is mediated by EZH2 (11). Expression of two cyclin-dependent kinase (CDK) inhibitors CDKN1A/p21 and CDKN1C/p57 were also demonstrated to be regulated by EZH2 in cervical and ovarian cancer, respectively (12,13). However, whether EZH2 participates in the regulation of Dicer expression has not yet been investigated.

In this study, we demonstrate the effect of Dicer downregulation on cell proliferation, cell migration ability and response to cisplatin in ovarian cancer *in vitro*. Furthermore, we reveal that EZH2 may be a key regulator of Dicer expression.

## Materials and methods

### Cell lines and culture conditions

Ovarian cancer cell lines A2780 and SKOV3 were purchased from the China Center for Type Culture Collection (CCTCC; Wuhan, China). A2780/DDP cells and stably EZH2-short hairpin RNA (shRNA)-transfected A2780 (shEZH2-A2780) cells were generated and reserved by our laboratory (13,23). All cell lines were cultured in Roswell Park Memorial Institute (RPMI)-1640 medium (Gibco; Carlsbad, CA, USA) with 10% fetal calf serum (FCS; Gibco), at 37°C in a humidified atmosphere of 5% CO_2_. The A2780/DDP cells used in the study were cultured in the absence of cisplatin for >1 month prior to use to exclude the stress reaction mediated by drug treatment.

The study was approved by the Ethics Committee of Huazhong University of Science and Technology, Wuhan, China

### Transient Dicer small interfering RNA (siRNA) transfection

Carboxyfluorescein (FAM)-labeled siRNA targeting Dicer and negative control siRNA were chemically synthesized (Invitrogen Life Technologies; Carlsbad, CA, USA). The siRNA sequences were as follows: Sense: 5′-UUUGUUGCGAGGCUGAUUCTT-3′ and anti-sense: 5′-GAAUCAGCCUCGCAACAAATT-3′ for Dicer siRNA; sense: 5′-UUCUCCGAACGUGUCACGUTT-3′ and anti-sense 5′-ACGUGACACGUUCGGAGAATT-3′ for negative control siRNA. Lipofectamine 2000 (Invitrogen Life Technologies) was used for transfection according to the manufacturer’s instructions. The transfection efficiency was detected by fluorescent microscopy and the growth medium was replaced after 6 h. Forty-eight hours after transfection, the cells were harvested for analysis.

### RNA isolation and quantitative polymerase chain reaction (qPCR)

Total RNA was extracted from the cell lines using TRIzol reagent (Invitrogen Life Technologies) according to the manufacturer’s instructions. Following confirmation of the quality and quantity of extracted total RNA by Nanodrop 2000 (Thermo Scientific, Wilmington, DE, USA), cDNA was synthesized using a reverse transcription kit (Toyobo; Osaka, Japan) according to the manufacturer’s instructions. The primer sequences for Dicer mRNA detection were as follows: Upstream: 5′-GTGGTTCGTTTTGATTTGCCC-3′ and downstream: 5′-CGTGTTGATTGTGACTCGTGGA-3′ (NM_001195573.1). The sequences of the β-actin primers were as follows: Upstream: 5′-GTCCACCGCAAATGCTTCTA-3′ and downstream: 5′-TGCTGTCACCTTCACCGTTC-3′. All reactions were performed in duplicate in an Applied Biosystems 7300 Real-time PCR system (Applied Biosystems; Foster City, CA, USA). Each reaction system contained 1 *μ*l cDNA sample, 12.5 *μ*l SYBR-Green Real-time PCR Master Mix (Toyobo) and 1 *μ*l of 10 *μ*mol/l each primer, in a final volume of 25 *μ*l. Reactions were performed under the following cycling conditions: Initial denaturation at 95°C for 1 min, followed by 40 cycles of denaturation at 95°C for 15 sec, annealing at 58°C for 15 sec and extension at 72°C for 45 sec. PCR products were identified by a melting curve analysis. The relative mRNA level of the Dicer gene was calculated using the comparative threshold cycle (Ct) method (2^−ΔΔCt^) normalized by β-actin expression (24).

### Protein extraction and western blot analysis

Protein for western blot analysis was isolated from cells by a radioimmuno-precipitation assay buffer (RIPA; Beyotime, China) according to the manufacturer’s instructions. The protein concentration was measured by bicinchoninic acid (BCA) assay. Fifty micrograms of total protein was denatured by boiling for 5 min, then separated by 10% sodium dodecyl sulfate-polyacrylamide gel electrophoresis (SDS-PAGE), transferred onto nitrocellulose membranes and blotted with mouse anti-Dicer (1:500 dilution; Abcam, Cambridge, MA, USA; Product No. ab14601) or mouse anti-β-actin (1:500 dilution; Santa Cruz Biotechnology Inc.; Santa Cruz, CA, USA). Primary antibodies were detected using horseradish peroxidase-conjugated anti-mouse secondary antibody (1:5000; Santa Cruz Biotechnology, Inc.) and visualized by an enhanced chemiluminescence kit (Pierce; Rockford, IL, USA). Protein bands were quantified following scanning by Quantity One software (Bio-Rad; Hercules, CA, USA).

### Cell proliferation assay

The cell proliferation assay was performed using a BrdU enzyme-linked immunosorbant assay (ELISA) kit (Calbiochem; San Diego, CA, USA) according to the manufacturer’s instructions. The absorbance at 450–595 nm was measured with an iMark microplate reader (Bio-Rad; Serial No. 10601).

### Cell viability assays

Cells were seeded in triplicate into 96-well plates (2000 cells/well) and then incubated with 3-(4,5-Dimethylthiazol-2-yl) -2,5-diphenyltetrazolium bromide (MTT) for 4 h, followed by dissolution in dimethylsulfoxide (DMSO) for 10 min every 24 h for 5 days. Growth curves were generated by calculating the mean value of the optical density measurements at 570 nm using the iMark microplate reader (Bio-Rad).

### Cell cycle analysis

Cells were harvested, trypsinized, washed with ice-cold phosphate-buffered saline (PBS) and fixed in 70% cold ethanol at 4°C overnight. Following centrifugation (1200 g for 5 min), fixed cells were washed with PBS, and then resuspended in PBS containing 1 mg/ml RNase A for 30 min at 37°C. Subsequently, cells were incubated with 50 *μ*g/ml propidium iodide for 30 min at 4°C. Cell cycle analysis was performed using a LSR flow cytometer (Becton Dickinson; San Jose, CA, USA) with ModFit LT software (Verity Software House; Topsham, ME, USA).

### Cell migration assay

The cell migration assay was performed using a Boyden chamber. Cells (1×10^5^/well) were trypsinized, resuspended in serum-free RPMI-1640 medium and then added to the transwell inserts (6.5 mm diameter, 8 *μ*m pore size, polycarbonate membrane; Corning Costar; Cambridge, MA, USA). RPMI-1640 medium (600 *μ*l) with 10% FBS was added to the lower chamber beneath the insert membrane. The transwell chambers were then incubated for 24 h under culture conditions. Migrated cells on the lower surface of the membrane were fixed with 70% ethanol and stained with crystal violet, and were then counted in 10 randomly selected high-power fields (×400) under a microscope. The average value was used as parameter to evaluate the migration ability of the cells. All assays were performed in triplicate.

### Drug cytotoxicity assays

Assessment of chemoresistance to cisplatin was determined by the MTT assays. Cells were seeded in triplicate at a density of 5000 cells/well in 96-well plates. Cells were treated with cisplatin at various concentrations ranging from 2.5–40 *μ*g/ml for an additional 24 h. Subsequently, 20 *μ*l of 5 mg/ml MTT was added to each well and, after 4 h, cells were dissolved in 150 *μ*l of DMSO for 10 min. The absorbance at 570 nm was measured using wells without cells as blanks on an iMark microplate reader (Bio-Rad; Serial No. 10601). The percentage of cell survival at each dose was calculated as the absorbance ratio of treated to untreated cells. The 50% inhibitory concentration (IC_50_) values were calculated by linear interpolation. Data shown are representative of three independent experiments.

### Statistical analysis

Data were expressed as mean ± standard deviation. The statistical significance of differences were estimated by a two-tailed Student’s t-test or a one-way analysis of variance (ANOVA), as appropriate, using the Statistical Package for the Social Sciences (SPSS) software, version 13 (SPSS Inc.; Chicago, IL, USA). P<0.05 was considered to indicate a statistically significant difference.

## Results

### Knockdown of Dicer by siRNA

**Figure 1:**
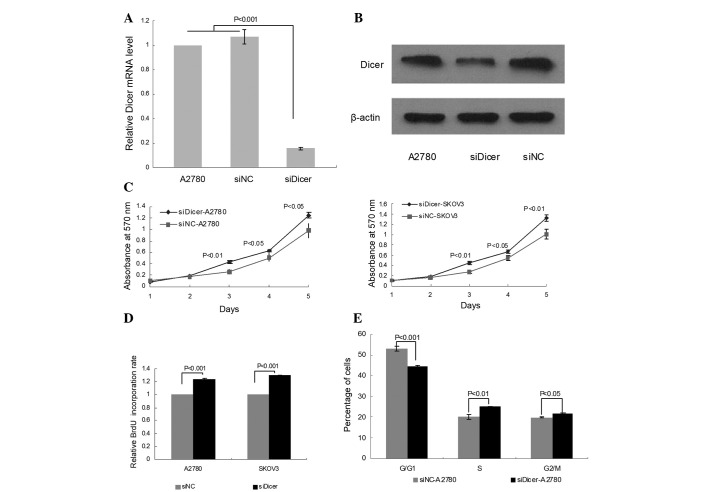
Transient transfection of Dicer small interfering RNA (siDicer) and effects of Dicer depletion on cell viability, proliferation and the cell cycle in ovarian cancer cells. (A) Expression of Dicer mRNA is detected by quantitative polymerase chain reaction (qPCR). The results are normalized to the expression of the reference gene β-actin. (B) Western blot analysis demonstrates a downregulation of Dicer protein in siDicer-A2780 cells. (C) Cell viability as evaluated *in vitro* by 3-(4,5-Dimethylthiazol-2-yl)-2,5-diphenyltetrazolium bromide (MTT) proliferation assays each day for 5 days following transfection. All data are representative of three independent experiments. (D) Cell proliferation as measured by a BrdU incorporation assay. The results are expressed as the fold change relative to the corresponding siNC-A2780/-SKOV3 control. Bars, standard deviation. (E) Cell cycle as analyzed by a propidium iodide flow cytometry assay. The percentages of S, G2/M and G0/G1 phase cells are calculated from three independent experiments. Bars, standard deviation.

To study the function of Dicer in ovarian cancer, transient Dicer-knockdown A2780 cells were generated using Dicer siRNA (siDicer). Untransfected A2780 cells and negative control siRNA (siNC)-transfected cells were used as controls. An 84.33% decrease in the level of Dicer mRNA was observed in siDicer-A2780 cells compared with untransfected A2780 cells by qPCR (P<0.001, Fig. 1A); whereas no significant difference was identified between siNC-A2780 and untransfected cells. The knockdown of Dicer was further confirmed by western blot analysis (Fig. 1B).

### Downregulation of Dicer promotes cell proliferation in ovarian cancer cells

To investigate the effect of Dicer knockdown on cell growth and proliferation in cancer cells, MTT and BrdU assays were performed for siDicer-transfected A2780 and SKOV3 cells. The MTT assay revealed that siDicer transfection significantly increased the cell viability of A2780 and SKOV3 on days 3, 4 and 5 compared with siNC (Fig. 1C). Additionally, the BrdU incorporation assay demonstrated that the proliferation of A2780 and SKOV3 cells was markedly stimulated by Dicer knockdown compared with siNC-transfected cells 96 h post-transfection (Fig. 1D).

To study whether the growth elevation upon Dicer depletion in ovarian cancer cells is associated with cell cycle regulation, cell cycle analysis was conducted by propidium iodide (PI) staining and flow cytometry. Dicer knockdown significantly increased the percentage of S and G2/M phase cells (P=0.002 and P=0.022, respectively), which was accompanied by a fall in the percentage of G0/G1 phase cells (P<0.001; Fig. 1E), suggesting that Dicer is a regulator of the cell cycle, impacting cell proliferation in ovarian cancer.

### Knockdown of Dicer promotes ovarian cancer cell migration

**Figure 2:**
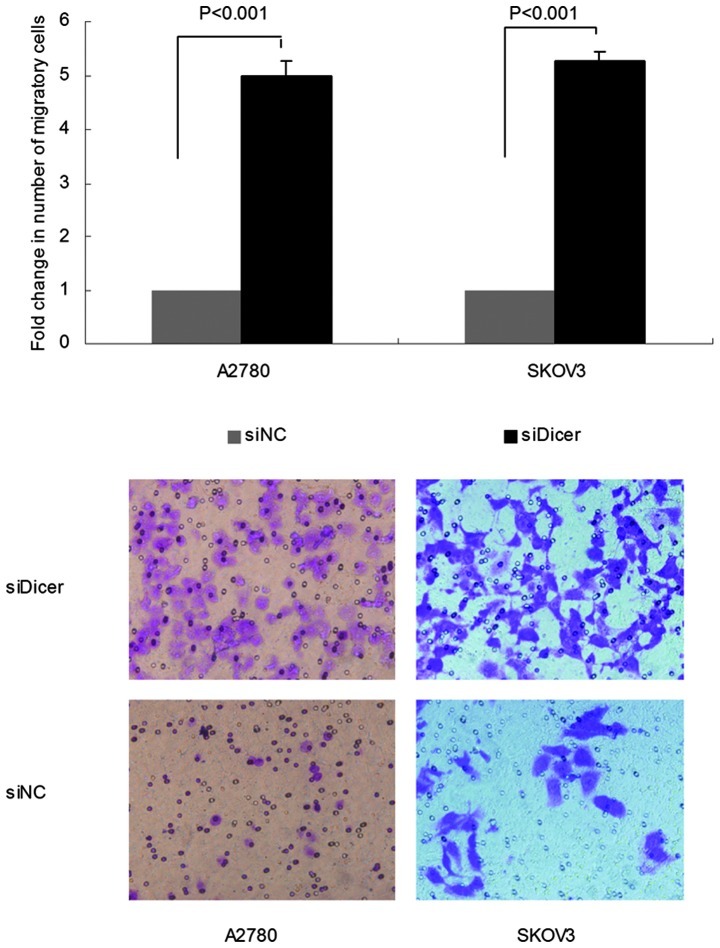
Effect of Dicer depletion on cell migration in ovarian cancer cells. Results of *in vitro* transwell migration assays are expressed by a histogram and are representative of microscopic images. Migrated cells on the lower surface of the membrane were stained with crystal violet and were counted in 10 randomly selected high-power fields under microscope. Bars, standard deviation.

Transwell migration assays for siDicer-transfected cells were subsequently performed. siDicer-A2780 and siDicer-SKOV3 cells exhibited an increased ability to migrate through an 8-*μ*m pore size polycarbonate membrane compared with siNC-transfected cells (P<0.001 for both cells; Fig. 2). The results thus far suggest that downregulation of Dicer in ovarian cancer may be required for disease progression.

### Downregulation of Dicer contributes to cisplatin resistance in ovarian cancer cells

**Figure 3:**
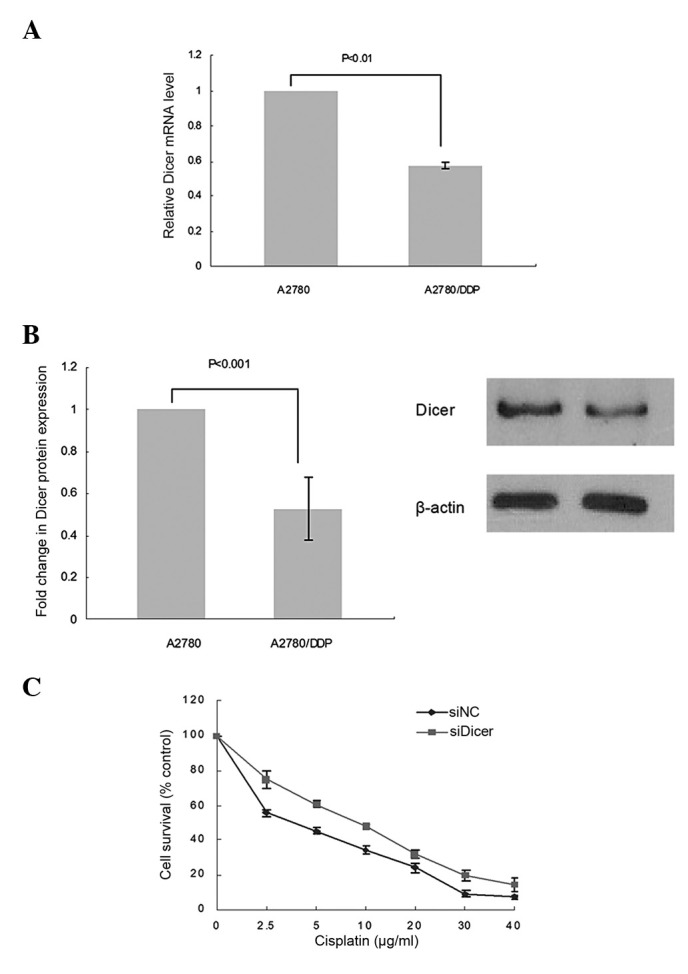
Downregulation of Dicer results in increased cisplatin resistance. (A) Quantitative real time-polymerase chain reaction (qRT-PCR) analysis reveals that the relative level of Dicer mRNA is decreased in A2780/DDP cells compared with A2780 cells. Dicer expression is normalized to β-actin. Bars, standard deviation. (B) Western blot analysis: Dicer protein expression is repressed in A2780/DDP cells compared with A2780 cells. Left: Histogram of quantified results of western blot analysis; right: Representative results of western blot analysis. (C) Results of 3-(4,5-Dimethylthiazol-2-yl) -2,5-diphenyltetrazolium bromide (MTT) assays. Cell survival following cisplatin treatment is significantly increased in Dicer small inerfering RNA (siDicer)-A2780 cells compared with negative control siRNA (siNC)-A2780 cells.

To delineate the role of Dicer in drug resistance, we first compared the expression of Dicer in A2780 cells and cisplatin-resistant cells derived from these (A2780/DDP) by qPCR and western blot analysis. A marked downregulation of Dicer expression was observed at both the mRNA (57.3% decrease; P<0.01; Fig. 3A) and protein (52.6% decrease; P<0.001; Fig. 3B) level in A2780/DDP cells compared with parental A2780 cells.

To verify the effect of Dicer knockdown on cisplatin sensitivity, the cell viability was assessed by MTT assays following treatment with various concentrations of cisplatin. Cell survival following cisplatin treatment was significantly increased in siDicer-A2780 compared with siNC-A2780 cells. Depletion of Dicer in the A2780 cells caused a 0.89-fold increase in the cisplatin IC_50_value (7.56 vs. 4.01 *μ*g/ml; P<0.01; Fig. 3C), indicating a causal correlation between Dicer repression and cisplatin resistance in ovarian cancer. However, further studies are required to clarify the underlying mechanism.

### Loss of EZH2 increased Dicer expression in ovarian cancer cell lines

**Figure 4:**
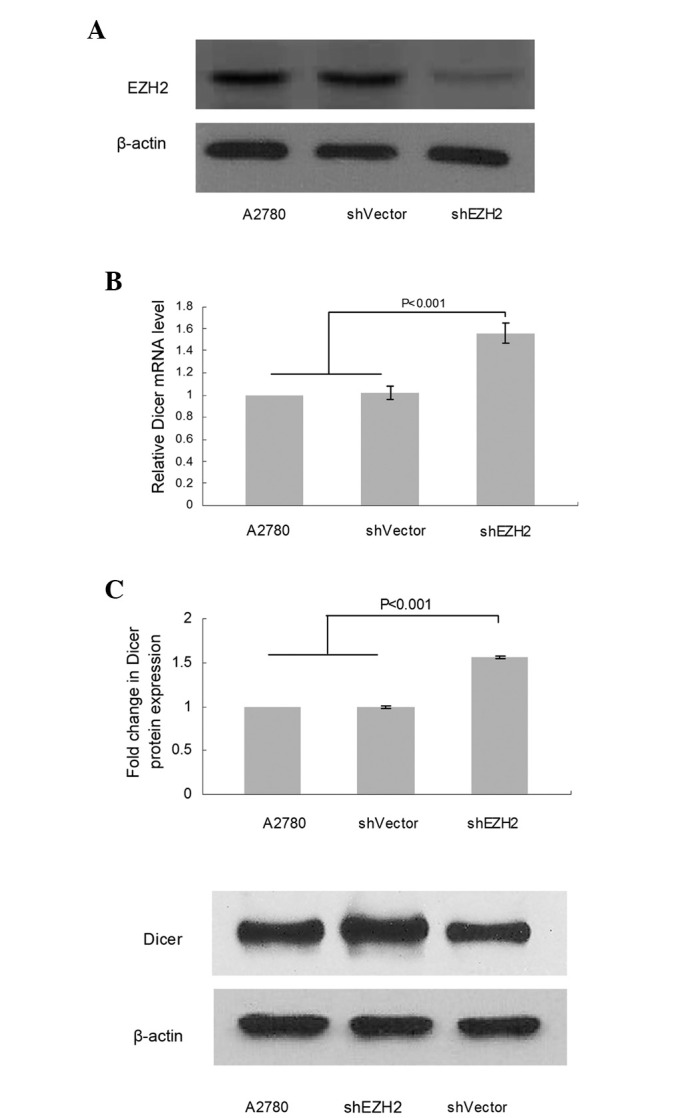
Loss of EZH2 increases Dicer expression in A2780 cells. (A) Western blot analysis shows downregulation of EZH2 protein in shEZH2-A2780 cells. (B) Quantitative real time-polymerase chain reaction (qRT-PCR). Dicer mRNA expression is elevated in short hairpin (sh) EZH2-A2780 cells compared with untransfected A2780 cells and shVector-A2780 cells. All data are representative of three independent experiments and normalized to β-actin. (C) Dicer protein is overexpressed in shEZH2-A2780 cells compared with untransfected A2780 and shVector-A2780 cells. β-actin, internal control. Bottom: representative western blot analysis of Dicer; top: histogram of quantified results of western blot analysis. All data are representative of three independent experiments.

To investigate whether EZH2 is involved in the regulation of Dicer expression in ovarian cancer, we analyzed the alteration in Dicer expression following EZH2 depletion mediated by shRNA (Fig. 4A) using qPCR and western blot analysis. qPCR revealed that the Dicer mRNA level increased by 55.7% in shEZH2-A2780 cells compared with NC (P<0.001; Fig. 4B) and a corresponding increase (56.0%) at the protein level was revealed by western blot analysis (P< 0.001; Fig. 4C). This result suggests that EZH2 is involved in the regulation of Dicer expression.

## Discussion

In the present study, reduced expression of Dicer in ovarian cancer was demonstrated to be associated with activated tumor cell proliferation, enhanced migration ability and increased cisplatin resistance. A number of studies have demonstrated similar effects of Dicer silencing in other types of cells. Dicer knockdown substantially increased the invasion ability of breast cancer cells (14) and the migratory capacity of human embryonic kidney (HEK) 293T cells *in vitro*(15). Moreover, at the molecular level, inhibition of Dicer in human cancer U251, MCF-7 and SCG7901 cell lines enhanced the expression of cell cycle-associating molecules cyclin A and PCNA, as well as invasion-promoting factors MMP-2 and MMP-9. Adenoviral gene silencing of Dicer in subcutaneous MCF-7 xenografts significantly increased the tumor growth *in vivo*(16). Kumar *et al* revealed that defective miRNA maturation enhanced tumor transformation and invasion *in vitro* and *in vivo*, and that conditional depletion of Dicer enhanced tumor development in a K-Ras-induced mouse model of lung cancer (17). Furthermore, Dicer was demonstrated to be required for proliferation, viability, migration and differentiation in corticoneurogenesis in a mouse model (18). A study on Dicer expression and function in ovarian cancer performed by Faggad *et al* indicated that decreased Dicer expression was significantly correlated with a global downregulation of the microRNA, advanced disease stages and reduced patient survival in serous tumors (19).

In accordance with the results of previous studies, the present study demonstrated that the reduced expression of Dicer in ovarian cancer is associated with activated tumor cell proliferation and enhanced migration ability. Additionally, for the first time, the role of Dicer in cisplatin resistance in ovarian cancer cells was investigated. Knockdown of Dicer in A2780 cells by siRNA was observed to promote cell cycle progression and to decrease sensitivity to cisplatin. A previous study had demonstrated that ablation of Dicer in the MCF-7 breast cancer cell line led to significant G1 arrest and increased sensitivity to cisplatin (20), suggesting that the role of Dicer in the regulation of the cell cycle and drug response is tumor type-specific. However, the effect on the invasion of Dicer silencing compared with that on cell survival and cisplatin resistance was observed to be more significant, suggesting that there are other factors besides Dicer affecting these pathways.

Although there are numerous studies concerned with Dicer, the regulation of its expression is poorly understood. Merritt *et al* measured the Dicer mRNA level in specimens of invasive epithelial ovarian cancer from 111 patients. Decreased Dicer expression was observed in 60% of cases. Mutational analysis in a subgroup of ovarian cancer specimens revealed rare missense mutations (2/37) in the Dicer gene, but its presence or absence was not correlated with the level of Dicer mRNA expression (9). Tokumaru *et al* demonstrated that let-7 miRNA inhibits the expression of Dicer, representing a negative feedback loop on overall miRNA production (21). Furthermore, Wiesen and Tomasi revealed that Dicer is post-transcriptionally regulated by cellular stresses and interferons (22). Previous studies demonstrated that EZH2 is upregulated in ovarian cancer and contributes to tumor progression and the development of cisplatin resistance *in vitro* and *in vivo*(13,23). To validate the regulation pathway and to explore the mechanism whereby EZH2 regulates Dicer expression requires further investigation.

In summary, we have demonstrated that loss of Dicer is capable of promoting cell proliferation, increasing cell migratory capacity and decreasing ovarian cancer sensitivity to cisplatin. Furthermore, for the first time, we provide evidence that implicates EZH2 in the regulation of Dicer expression. Further investigation into the function of Dicer in carcinogenesis and its regulation pathways in human ovarian cancer tissue, additional cell lines and animal models will promote our exploitation of novel anti-cancer targets.
